# The Role of Silicon Compounds in Plant Responses to Cadmium Stress: A Review

**DOI:** 10.3390/plants14182911

**Published:** 2025-09-19

**Authors:** Monika Komorowska-Trepner, Katarzyna Głowacka

**Affiliations:** Department of Plant Physiology, Genetics and Biotechnology, Faculty of Biology and Biotechnology, University of Warmia and Mazury in Olsztyn, 10-719 Olsztyn, Poland; monika.komorowska-trepner@uwm.edu.pl

**Keywords:** metasilicates, nanoparticles, ROS, subcellular changes, metal chelation

## Abstract

Silicon (Si) has emerged as a promising tool for mitigating the negative effects of biotic and abiotic stresses, such as caused by heavy metals, on plants. The aim of the study was to summarize knowledge about the mechanisms underlying the interaction between silicon and cadmium. This review first discusses silicon compounds in plant physiology, then examines mechanisms of silicon–cadmium interaction, including antioxidant defense, metal chelation, nutrient transport, molecular responses, subcellular changes, and future directions. Recent studies show that various forms of Si, such as conventional Si and Si-nanoparticles (Si NPs), can have various effects on the ability of a plant to absorb and utilize Si for protection. Silicon, taken up mainly as soluble orthosilicic acid (H_4_SiO_4_) and Si NPs, can be absorbed by plants and subsequently deposited predominantly in cell walls. It has been found that Si and Si NPs increase the activity of antioxidant enzymes, including CAT, SOD, and POD, in plants under cadmium (Cd) stress. Furthermore, Si reduces the expression of Cd transport-related genes, including *OsNRAMP5* and *OsHMA2* in rice. It has also been shown that supplementation with Si and Si NPs in plants under Cd stress reduces the Cd content in their tissues and changes the uptake of elements necessary for the proper functioning of the plant organism. Furthermore, Si supplementation increases the content of pectins, which are involved in the binding and neutralization of Cd. The following overview highlights the importance of both Si and SiNPs in neutralizing the harmful effects of Cd on the environment and agriculture.

## 1. Introduction

Heavy metal pollution resulting from industrial activities is a serious environmental problem. According to the definition given by Ali and Khan [1], heavy metals are elements that are naturally occurring metals with an atomic number (Z) greater than 20 and an elemental density greater than 5 g cm^−3^. Heavy metals are often referred to as elements that cause toxic environmental effects. However, as highlighted by Duffus [2], this term is imprecise, and it is suggested that it be replaced by classifications based on chemical properties and bioavailability.

Cadmium (Cd) is one of the most toxic heavy metals. Industrial activities, which mainly include mining, metallurgy, or waste disposal, wastewater irrigation, Ni-Cd battery production, and electroplating, lead to the release of Cd into water and soil [3,4]. At the same time, there are natural pathways for the release of Cd into the environment, including volcanic eruptions, forest fires, and rock weathering. Particularly high Cd release into the environment persisted in the 1940s, when intensive industrial development was carried out around the world [3].

According to the World Health Organization (WHO), the permissible weekly intake of cadmium (Cd) for humans is 7.00 µg kg^−1^ body weight; in turn, exceeding 75.00 µg day^−1^ is considered a health hazard. The use of phosphate fertilizers has also been found to significantly increase the accumulation of Cd in cultivated soils [5]. Under European Union (EU) Regulation 2019/1009, the permissible cadmium content in phosphate fertilizers must not exceed 60 mg Cd per kg P_2_O_5_ [6]. Ballabio et al. [7] analyzed 21.682 topsoil samples of the European Union and the UK, showing that 5.5% of samples exceeded 1 mg kg^−1^. Moreover, the average cadmium content in samples from the EU was 0.20 mg kg^−1^, while the average Cd content in farmland was 0.17 mg kg^−1^. For example, the lowest permissible Cd concentration in soil in Poland is 2 mg kg^−1^ of dry weight [8]. However, exceedances of the standards are still encountered [9].

Cd pollution is a serious problem because of this element’s high mobility, high solubility in water, and the ability to bioaccumulate in plant tissue cells [10,11]. Cadmium, which bioaccumulates in edible parts of plants, enters the food chain. Plants contaminated with Cd are consumed by humans and animals, which is a factor implicated in the etiology of numerous diseases, such as osteoporosis, osteomalacia, rheumatoid arthritis, and cancer [10,12,13,14]. Diseases of the skeletal system are mainly due to cadmium’s high affinity for calcium (Ca), facilitating the substitution of these elements inside bone structures [10]. Cadmium is also a causative factor in liver and kidney diseases [15].

Cadmium can enter plant organisms via three pathways. The first pathway is the uptake of Cd through transmembrane carriers located in plant root cells, which are designed for micro- and macroelements. Carriers for calcium (Ca), iron (Fe), zinc (Zn), and copper (Cu) play an important role in this process. The uptake of Cd by these carriers contributes to deficiencies in elements necessary for the proper functioning of the plant organism, which affects the functioning of many biological pathways in plant tissues [16].

The second pathway through which Cd can enter the plant is the passive pathway associated with respiration. During the respiration process in plant roots, carbon dioxide (CO_2_) is released into the soil solution, which then dissolves in water and forms carbonic acid (H_2_CO_3_). This acid subsequently hydrolyzes to bicarbonate ions (HCO_3_^−^) and hydrogen ions (H^+^). The acidification of the rhizosphere due to the release of H^+^ ions promotes the desorption of Cd^2+^ ions from soil particles, making them more available for uptake. In the passive pathway, Cd^2+^ ions are adsorbed on the surface of root cell walls and can be absorbed in exchange for H^+^ ions, maintaining the Donnan equilibrium in the apoplast. From there, cadmium can continue to move within the plant via the apoplastic pathway (along cell walls and intercellular spaces) without entering the cell cytoplasm [16]. The third pathway through which Cd can enter the plant organism is related to substances released by plant root tissues into the soil solution, which are designed to increase the availability of essential ions. Among these substances are mugineic acids (MAs), which can also chelate Cd^2+^ ions. The resulting complexes are taken up by root cells through Yellow Stripe-Like (YSL) transport proteins located in the rhizodermis. However, these chelates typically dissociate before reaching the endodermis. Only free Cd^2+^ ions are actively transported across the endodermal barrier via specific carrier proteins in an energy-dependent manner [17].

The above-mentioned pathways of Cd release into the environment and its penetration into plant tissues are not only the cause of diseases in humans and animals consuming Cd-contaminated plants but can also impair many physiological and biochemical functions of the plants themselves. This leads to a reduction in plant production by lowering crop yields. It was found that the bioaccumulation of Cd in plant tissues leads to several adverse effects, e.g., disruption of mineral transport, reduction in chlorophyll content, inhibition of photosynthesis, and damage to proteins and DNA [11,18,19,20,21]. It was shown that Cd can replace magnesium (Mg) in the porphyrin ring of the chlorophyll molecule, which impairs its functions and prevents the proper course of photosynthesis [22]. Wahid et al. suggested that Cd may inhibit CO_2_ binding by Rubisco, a key enzyme involved in photosynthesis [23]. It was also shown that treatment with a 1 mM Cd solution resulted in a statistically significant reduction in the rate of photosynthesis in peas by 16.7% and in barley by 12.8% compared to the control plants [24]. Cadmium also contributes to plant growth inhibition, reduction in water content in plant tissues, and reduction in biomass [25]. Table 1 shows the impact of Cd on various plant species.

Plants under Cd stress activate a number of defense mechanisms leading to its neutralization. Among the defense mechanisms of plants is the restriction of transport of this element to the aerial plant parts by creating physical barriers that inhibit apoplastic transport to the xylem (suberin, lignin, cellulose, silica), or the synthesis of sulfur-containing chelates (such as phytochelatins) that bind Cd and are then stored in vacuoles [33,34,35,36].

The numerous negative effects of Cd on plant tissues have prompted the studies on ways to neutralize them. Silicon (Si) has proven to be particularly effective in alleviating Cd stress. Silicon is the second most abundant element in the world, accounting for 50–70% of soil mass, and it is also a component of plant tissues [37].

To date, numerous review papers have been published focusing on the effect of Si or Si NPs on plants under cadmium stress [38,39,40]. These reviews have emphasized analyses covering a specific plant species, e.g., rice [38], or focused on summarizing a wide range of heavy metals, including arsenic (As), aluminum (Al), lead (Pb), and cadmium [39]. Furthermore, existing review papers cover the impact of Si or Si NPs on plants under cadmium stress, but there is a lack of papers summarizing the impact of both compounds [40]. There have also been analyses focusing on the interaction of mycorrhizal fungi (MF) and silica-solubilizing bacteria (SSB) in Cd-treated plants, with the simultaneous use of Si [41]. In contrast to the previously cited review studies, this summary addresses a wide spectrum of plant species and integrates findings on both Si and Si NPs under cadmium stress conditions. Particular attention is given to the differences between the application of soluble forms of Si (mainly orthosilicic acid) and silicon nanoparticles (Si NPs). In recent years, nanotechnology and the use of nanoparticles in plant physiological studies have attracted increasing interest, especially with regard to their potential role in alleviating symptoms of abiotic stress, although the underlying mechanisms are not yet fully understood. In the following sections, we describe the effects of Si on the physiological responses of plants treated with Cd, including its influence on Cd uptake and accumulation by downregulation of Cd transporters expression, as well as its binding and compartmentalization within cells, particularly in cell walls and vacuoles due to increased pectin content or phytochelatin synthesis, respectively.

## 2. Silicon Compounds in Plant Physiology

Plants take up Si found in soil solution (at pH < 9) in the form of soluble monosilicic acid: Si(OH)_4_ [(H_4_O_4_Si)] [42,43]. Silicon is most often applied to plants directly to the soil or as foliar sprays [44,45]. The mode of uptake of this element by plants was shrouded in mystery until the authors Ma et al. [46] first described the protein silicon transporter Lsi1 (*low silicon rice 1*) in rice plant mutants that lacked the ability to take up silicon. These studies showed that Lsi1 belongs to the aquaporin family, and silicon is taken up without energy input in the form of ATP. A year later, the Lsi2 (*low silicon rice 2*) transporter was also characterized as being responsible for pumping silicon out of the cell interior via a proton gradient [47]. In the aerial parts of plants, silicon undergoes compaction and polymerization as a result of transpiration, ultimately leading to the formation of colloidal silica gel [42]. To date, the process of silicon transport and deposition in the aerial parts of plants has been analyzed in many studies. Intensive silicon deposition was first demonstrated in the endodermis, the inner part of the primary cortex of rice (*Oryza sativa* L.) plants. It was also found that plant species differ in their ability to deposit silicon in specific organs [48]. Jones and Handreck suggested that plants of *Trifolium incarnatum* L. have a barrier located in the roots through which monosilicic acid is transported more slowly than water. In addition, the silica content contained in the tops of the analyzed plants was lower than in the root, despite increasing concentrations of silica present in the solution in which the plants were grown [49]. In turn, Ma and Takahashi [50] showed that individual plant species differ in their ability to bioaccumulate silicon, among which grasses [*Poaceae* (R. Br.) Barnh.] and sedges (*Cyperaceae* Juss.) have the highest ability to accumulate this element.

Many studies showed positive effects of Si on plant organisms, but it is important to emphasize the fact that they are mainly manifested in the presence of stress factors [51]. Silicon benefits plants under drought, salt stress, or cadmium stress. It has been demonstrated that phytoextraction, which involves the binding of heavy metals with phytoliths consisting of amorphous silica, e.g., in plant cell walls, plays an important role in plant tolerance to stress caused by the presence of heavy metals and reduces the content of heavy metals in the soil solution [52]. Lux et al. [53] showed that sorghum (*Sorghum bicolor*) plants of the Gadambalia variety, grown around Sudan and characterized by high drought tolerance, have characteristic anatomical adaptations that enable them to survive under adverse environmental conditions. These include increased amounts of silicon deposited in the endodermis of root cells. The authors showed that this represents a difference from the Tabat sorghum variety, which exhibits less tolerance to drought. On the other hand, Gao et al. [54] found that the application of 2 mmol L^−1^ silicic acid to maize plants reduced the transpiration process and suggested that Si may affect the movement of stomatal apparatuses. Silicon may also affect the neutralization of stress resulting from salinity. It was shown that treatment of sweet basil (*Ocimum basilicum* L.) under salt stress (induced by the presence of 6000 mg NaCl per kg soil) plants with 100 mg L^−1^ Si improved, among other things, osmotic homeostasis and antioxidant capacity of the plants analyzed [55]. Silicon was also shown to have a positive effect on neutralizing the stress caused by the presence of cadmium, manifested in a reduction in the uptake and accumulation of this heavy metal [56,57,58]. Despite a number of studies indicating positive effects of Si on Cd-treated plants, there are conflicting reports that present different hypotheses relating to the role of silicon in plant organisms. One of these is ‘the apoplastic obstruction hypothesis’, which points to an indirect role for this element in alleviating plant stresses, inter alia by strengthening physical barriers in the apoplasm, and inhibiting the transport of undesirable substances such as heavy metals into the aerial parts of plants [59].

Nowadays, nanoparticles (NPs) and their effect on neutralizing plant stresses are of increasing interest to researchers. Nanoparticles, due to their small size (from 1 nm to 100 nm), exhibit a number of specific physicochemical properties, so they have broad application potential in electronics, medicine and agriculture, among others [60]. Nanoparticles of silicon, one of the most common elements in the world, and silicon oxide (SiNPs; SiO_2_NPs) are considered relatively non-toxic and safe. There are three ways to apply nanoparticles: as a foliar spray, seed treatment (nanopriming), and direct application to the soil [61]. The routes of penetration of SiNPs/SiO_2_NPs into the plant organism depend on where they are applied. Nanoparticles applied as a foliar spray are transported into the plant organism through the leaf epidermis or stomata. When nanoparticles are applied to the soil solution, they penetrate the roots by forming complexes with substances released into the soil (organic acids, amino acids), which can then combine with transmembrane proteins and penetrate the root tissues through ion channels, among others [62]. It was found that nanoparticles can also enter the plant via the Lsi1 transporter, while due to their small size, they more easily cross cell membranes [63]. Silicic acid taken up from the soil solution is further transported into exodermal cells by passive transporters Lsi1. It then leaves the exodermal cells via Lsi2 transporters, whose function depends on a proton gradient maintained by a proton pump. From the apoplastic space of the aerenchyma, silicic acid moves further into the endodermal cells, where it is again taken up by Lsi1 and transported into the interior of these cells. From the endoderm, it is then exported via Lsi2 transporters towards the xylem. The process of silicic acid loading into the xylem has not yet been fully elucidated and is believed to occur passively, although the existence of an additional yet unknown transporter responsible for this process is possible [64].

Absorption of silicon nanoparticles from the environment to plant cells can occur via active and passive absorption. Using a solution of silicon nanoparticles as a foliar spray as well as direct application to roots enables their permeation into plant tissues. The process of passive absorption is determined by the small size of SiNPs, which enables nanoparticles to enter directly into plant tissues (bypassing silicon transporters) through stomata and the cuticle as well as through immature elements of root tissues. Having entered root cells, SiNPs can be transported both apoplastically and symplastically towards the xylem cells. Moreover, silicon nanoparticles can be transformed in the soil solution to silicic acid, which penetrates the inside of root cells with the help of Lsi1 transporters (passive transport) or else it is transported towards the xylem using Lsi2 transporters (active transport) [65]. SiNPs translocate to the inside of xylem cells by binding to carrier proteins or by creating pores in xylem cel walls. The process of translocation of nanoparticles depends on their charge, hydrophobicity, and size. Nanoparticles which have been transported to aerial plant organs can accumulate inside plant cells (including wall cells, the cytoplasm). Nanoparticles can also accumulate in plant organs that serve to accumulate plant juices, roots, leaves, fruit, and seeds of plants [66].

The results of many studies showed the positive effect of nanoparticles on neutralizing the negative effects of stress caused by drought, salinity, and the presence of heavy metals in plants [67,68,69]. Both silicon and silicon nanoparticles displayed a number of positive properties in plants under various stress factors, especially cadmium. There are also studies that compare the effects of nanoparticles and silicates on plant organisms. It was found that the application of both 2.5 mM Na_2_SiO_3_ and 2.5 mM SiO_2_NPs to *Trigonella foenum graceum* L. plants did not result in statistically significant differences in antioxidant enzyme content and protein content between plants treated with silicate and nanoparticles. It was also demonstrated that the application of Si can result in an increase in soil pH and changes in the soil Cd fractions. For instance, the application of 0.8 g Na_2_SiO_3_·9H_2_O to soil contaminated with 1.83 mg kg^−1^ Cd induced a decrease in the Cd content in fraction Exc-Cd (easily exchangeable fraction) and an increase in the Cd content in fractions OX-Cd (fraction bound to iron and manganese oxides) and OM-Cd (fraction bound to organic matter), which suggests that Si affects the translocation of Cd from easily available, less stable soil fractions [70]. Depending on the soil pH, silicon can display varied effects on the uptake of cadmium by root plants. It has been demonstrated that in acidic soils, the addition of silicon reduces the absorption of cadmium by plants, which is a consequence of the increased binding of Cd in soil. In alkaline soils, Si affects the formation of Si-Cd complexes, unavailable to plants [71]. Furthermore, it has been determined that a dose of 1000 kg ha^−1^ K_2_SiO_3_ added to clayish, sandy, alum shale, and submerged soils increased the availability of Ca, P, S, Mn, Zn, Cu, and Mo, although no statistically significant differences were observed in cation exchange capacity (CEC) [72].

It was also shown that both forms affected cell wall lignification. On the other hand, the application of Na_2_SiO_3_ increased the expression of the *PST* transporter gene and the transport of silicon into the shoots of the analyzed plants. *PST* (putative silicon transporter) is a gene presumed to encode a protein involved in the uptake and translocation of silicon within plant tissues. The application of SiO_2_NPs, on the other hand, increased the total silicon content of the plant, but resulted in less efficient transport of Si to the aerial parts of the plant [73].

This article is a literature review summarizing the state of knowledge to date on the effects of silicon and silicon oxide nanoparticles on plants under cadmium stress, which includes their effects on antioxidant activity, nutrient transport, proteome, transcriptome, and subcellular changes in plants.

## 3. Mechanisms of Silicon–Cadmium Interaction in Plants

### 3.1. Antioxidant Protection and Oxidative Stress

Cadmium stress contributes to the formation of large amounts of reactive oxygen species (ROS) in plant tissues [74,75]. This element does not participate directly in oxidation and reduction reactions but impairs the action of antioxidant enzymes of plants, resulting in the accumulation of ROS, any excess of which leads to cellular damage [76]. ROS include hydroxyl radicals (·OH), superoxide anions (·O_2_^−^), hydrogen peroxide (H_2_O_2_), singlet oxygen (^1^O_2_), hydroperoxide radical (HOO·), alkoxyl (RO·), and radical peroxyl (ROO·) [77,78]. In turn, plant antioxidant enzymes that neutralize ROS include superoxide dismutase (SOD), catalase (CAT), peroxidases (POX), glutathione peroxidase (GPX), glutathione reductase (GR), and ascorbate peroxidase (APX) [78]. The above enzymes have many important functions in plant cells. Catalase is involved in the conversion of H_2_O_2_ to H_2_O and O_2_. Glutathione peroxidase is involved in neutralization of H_2_O_2_ and HO_2_ by catalyzing the reduction in these compounds to water and lipid alcohols. The APX enzyme, on the other hand, is involved in the reduction in H_2_O_2_ to H_2_O. The GR enzyme is involved in the reduction in oxidized glutathione (GSSG), a reduced form of glutathione that plays an important role in neutralizing ROS which contributes to the renewal of GSH reserves. When GSH neutralizes ROS, it becomes oxidized to GSSG, which is why it is important to renew GSH reserves through GR activity [78]. In addition, MDA (malondialdehyde), which is a product of lipid peroxidation conducted through ROS, is another indicator of oxidative damage to monitor the level of cellular damage [79].

Silicon applied both in the form of metasilicates and nanoparticles affects the antioxidant system of cadmium-treated plants. The most frequently observed response is an increase in the activity of antioxidant enzymes under the influence of Si compared to the level recorded after cadmium treatment. Such results have been described after application of silicon as both a soil additive and foliar sprays, as well as in studies conducted under hydroponic conditions.

Methosilicates or monosilicic acid applied to the soil were used during studies conducted on pearl millet [80] and wheat [81]. The results of these analyses indicated that the application of 200 mg kg^−1^ CdCl_2_ increased the activity of SOD, POD, APX, and CAT compared to plants treated with CdCl_2_ alone [73]. In contrast, the application of 1 mM Na_2_O_3_Si·9H_2_O to *Triticum aestivum* plants treated with cadmium (200 µM CdSO_4_·8H_2_O) increased APX, GR, and CAT enzyme activities in the roots compared to plants under cadmium stress [81]. Similar results were obtained after soil application of SiNPs. Ahmed et al. [69] conducted an experiment to evaluate the effect of bulk Si and SiNPs on rapeseed plants grown in pots with the addition of 3.5 mg kg^−1^ Cd(NO_3_)_2_. The authors showed that the application of Cd caused a statistically significant decrease in the activity of antioxidant enzymes SOD, POD, CAT, and APX in rapeseed leaves compared to the control sample. On the other hand, the addition of Si at concentrations of 100 and 250 mg kg^−1^ to Cd-contaminated soil resulted in a statistically significant increase in the activity of SOD and POD enzymes compared to Cd-treated plants. However, the application of SiNPs at a concentration of 250 mg kg^−1^ in Cd-contaminated soil increased the activity of antioxidant enzymes (SOD, POD, CAT, and APX) compared to plants grown in soil with Cd. Similar results were obtained during studies conducted on rice (*Oryza sativa* L.). Faisal et al. [82] grew rice in soil contaminated with 0.8 mM CdCl_2_ after silicon application (immersion of roots for 15 min in 100 mg L^−1^ SiO-NPs). They observed an increase in SOD, CAT, and POX activity, an increase in proline content, and a decrease in the content of MDA and H_2_O_2_ compared to plants treated with Cd.

The results of experiments conducted under hydroponic conditions also confirmed an increase in the activity of antioxidant enzymes after the application of metasilicates as well as SiNPs. Hasanuzzaman et al. [83] in their work analyzed the effect of 1 mM SiO_2,_ 1 mM CdCl_2_ and the combined interaction of the two compounds on rapeseed. The results of the experiment showed that plants treated with cadmium and silicon had higher APX enzyme activity compared to plants treated with cadmium. The application of 150 mg L^−1^ CdSO_4_·8H_2_O to pea (*Pisum sativum* L.) increased CAT activity compared to the control by 28.96%, while the addition of 2 mM Na_2_SiO_3_ increased the activity of this enzyme by an additional 41.45% [84]. On the other hand, in the work of Azam et al. [85], treatment with 2 mM Na_2_SiO_3_ of *Isatis cappadocica* and 600 μM CdCl_2_ increased GST activity compared to plants treated with Cd. In addition, the combined effect of these compounds did not cause significant differences in the amount of H_2_O_2_ in the shoots of the analyzed plants, while the application of 1 mM Na_2_SiO_3_ in plants treated with Cd induced a statistically significant reduction in hydrogen peroxide content compared to plants treated with cadmium. Moreover, analysis of the effect of 0.6 mM monosilicic acid and 15 μM CdCl_2_·H_2_O on wheat plants showed an increase in the activity of CAT and APX enzymes compared to plants treated with Cd alone [86]. In contrast, in another study, treatment of *Pfaffia glomerata* plants with 2.5 mM Na_2_SiO_3_ and 500 μM CdCl_2_ increased the activity of SOD and POX enzymes in shoots and roots of the analyzed plants compared to plants treated with cadmium [87]. The stimulating effect of Si on the activity of antioxidant enzymes was also confirmed in a study where 1 mmol L^−1^ silica gel was applied to the roots of *Triticum aestivum* exposed to 200 μmol L^−1^ CdCl_2_. This treatment increased the activity of CAT, SOD, and POD enzymes compared to Cd-treated plants [88]. On the other hand, Zhou et al. [89], through analyses conducted on soybean plants, showed that the application of 1.5 mM Na_2_SiO_3_·9H_2_O to plants treated with 20 μM CdCl_2_ increased the activity of SOD and POD enzymes in the roots of the analyzed plants compared to plants treated with Cd. Interestingly, the authors showed that there were no significant differences in SOD and POD activity between the cited variants in the shoots of the analyzed plants.

Silicon nanoparticles are also important in neutralizing cadmium stress when applied under hydroponic conditions, as confirmed by numerous studies. A study on wheat conducted by Rahman et al. [90] showed an increase in the activity of enzymes CAT, SOD, and POD in the roots of the analyzed plants treated with 200 µmol L^−1^ CdCl_2_ compared to the control sample (without the addition of Cd). In contrast, the application of 3 mmol/L silicon nanoparticles together with Cd increased the activity of these enzymes compared to plants treated with 200 µmol L^−1^ CdCl_2_ alone. Ashraf et al. [91] found that the application of 50 μM SiO_2_NPs and 100 μM CdCl_2_ in *Oryza glumaepatula* plants caused a statistically significant increase in activity of SOD, POX, CAT, and GR compared to plants treated with Cd alone, while the application of SiO_2_NPs reduced MDA content by 24% compared to plants treated with CdCl_2_. Similar data were also obtained by Malik et al. [92] on melon (*Cucumis melo*). In this work, the authors treated melon seedlings with cadmium and silicon oxide nanoparticles. The results confirmed that the application of SiO_2_NPs at a concentration of 75 mg L^−1^ together with 100 μM Cd increased APX activity and reduced MDA content compared to plants treated with cadmium alone. An increase in MDA content in plant tissues after cadmium stress was also observed by Yan et al. [93]. In this work, the authors analyzed the effects of silicon and silicon nanoparticles on tomato (*Solanum lycopersicum* L. cv. Microtom) seedlings. They showed that 100 μM Cd(NO_3_)_2_ reduced the activity of the SOD and CAT compared to a control plants. On the other hand, the application of 2 mM Na_2_SiO_3_ or 2 mM SiNPs to plants exposed to cadmium stress contributed to an increase in SOD activity in roots and shoots compared to plants treated with cadmium. Interestingly, the application of these compounds did not increase CAT activity compared to plants treated with Cd. In contrast, Cd-stressed plants showed higher APX activity in roots and shoots compared to the control plants, while the application of SiNPs reduced the activity of this enzyme in roots and shoots of the tomato seedlings. Moreover, Sun et al. [94] analyzed seedlings of *Momordica charantia* L. plants. The analyses conducted by the above authors showed that the application of 50 μmol L^−1^ CdCl_2_ and 30 mg L^−1^ nSiO_2_ increased the activity of the APX enzyme in the roots, stems, and leaves of the analyzed plants compared to plants treated with cadmium alone.

On the other hand, the work of Jalil et al. [95] conducted on rice seedlings showed that treatment of plants with 20 µM L^−1^ CdN_2_O_6_·4H_2_O and 50 and 100 mg L^−1^ SiO NPs caused a statistically significant reduction in the content of MDA and H_2_O_2_ in the shoots of the analyzed plants compared to plants subjected to cadmium stress alone. In addition, it was shown that the combined application of these compounds did not increase the activity of SOD, POD, and CAT whose activity was statistically significantly lower compared to plants treated with cadmium alone, a finding that differs from the data presented in other reviewed works. The application of silicon, in some plant species, at low concentrations can affect the decrease in the activity of antioxidant enzymes. This relationship was observed in the study of He et al. [96], where the effect of nano-silicon dioxide (nSiO_2_) on seedlings of barley (*Hordeum vulgare* L.) plants subjected to cadmium stress was analyzed. Interestingly, the authors of the above work showed that treatment of barley seedlings with 50 μM CdCl_2_ increased the activity of the SOD, APX, CAT, and POD, while additional treatment with nSiO_2_ at concentrations of 5, 10, 20, and 40 mg L^−1^ decreased the activity of these enzymes compared to plants treated with cadmium alone. At the same time, as the concentration of nanoparticles increased, enzyme activity increased again, reaching its highest value at 40 mg L^−1^ nSiO_2_.

Silicon can also be applied as foliar spraying. Alamri et al. [97] analyzed the effect of such Na_2_SiO_3_ application on flax plants of the cultivars Canadian Camelina and Australian Camelina under cadmium stress induced by the presence of 5 ppm CdCl_2_. Both varieties of Cd-treated flax plants resulted in a statistically significant increase in the activity of antioxidant enzymes APX, CAT, POD, and SOD compared to plants treated with CdCl_2_ alone. El-Okkiah et al. [98] also analyzed the effect of silicon on pea plants treated with cadmium. In their work conducted in 2018/2019 and 2019/2020, they showed that pea plants treated with 100 mg kg^−1^ Cd and 300 ppm Si as a foliar spray showed reduced MDA content compared to plants treated with Cd alone. In addition, the application of Si increased the activity of POD and CAT enzymes. Moreover, Thind et al. [99] also found a neutralizing effect of silicon on cadmium-stressed wheat plants of cultivars Sahar-2006 and Inqalab-91. They showed that the application of a foliar spray of silicon in the form of 3 mM Na_2_SiO_3_ on plants treated with 10 mg kg^−1^ CdCl_2_ reduced the MDA and H_2_O_2_ content in the leaves of the studied plants. In addition, they showed that the application of Si foliar spray increased the activity of SOD, POD, CAT, and APX compared to plants treated with cadmium. Another study also showed that a foliar spray application of 4.50 mM potassium silicate on wheat plants exposed to 20 mg kg^−1^ CdCl_2,_ increased the activity of SOD, POD, and CAT [100]. The neutralizing effect of silicon on cadmium-stressed plants was also demonstrated in basil (*Ocimum basilicum* L.) [101], rice (*Oryza sativa* L.) [102], and corn (*Zea mays* L.) [103].

Foliar application of nanoparticles also markedly affects the activity of antioxidant enzymes of Cd-treated plants. Analysis of the effect of 2.5 mM SiNPs as a foliar spray on rice seedlings treated with 50 μM CdCl_2_ showed an increase in GSH content and APX activity compared to plants treated with Cd. In the experiment presented here, plants treated with Cd also showed an increased MDA content of 229.86% compared to the control plants, while the application of silicon nanoparticles to plants exposed to cadmium stress induced a decrease in MDA content of 56.47% compared to plants treated with cadmium [104].

The above results suggest that both silicon and silicon nanoparticles show a neutralizing effect on the cadmium stress in various plant species. An increase in the activity of antioxidant enzymes in plants exposed to cadmium and treated with silicon indicates an important role of this element in plant defense mechanisms. In addition, the reduction in MDA, an important indicator of lipid peroxidation that can lead to damage of the cell membranes, caused by silicon application further confirms positive effect of Si in antioxidant mechanisms. Numerous studies showed an increase in the activity of CAT, POD, and SOD, among others, following the application of silicon or silicon nanoparticles in plants under stress, further indicating the important role of silicon in the processes of alleviating cadmium stress in plants.

Interestingly, Miao et al. [105] suggested that the antioxidant changes observed after treating plants with SiNPs may vary depending on the size of the nanoparticles used. Nanoparticles with a diameter of 3–5 nm are characterized by their ability to penetrate cells through osmotic forces, while nanoparticles with a diameter of 10–20 nm are retained in the intercellular spaces of leaves. The authors suggested that small-diameter nanoparticles penetrating cells may interfere with cytoplasmic antioxidant systems. In contrast, larger nanoparticles can inhibit antioxidant enzymes, resulting in the accumulation of ROS in the apoplasm, which leads to the activation of salicylic acid (SA)-dependent defense pathways and the enhancement of intracellular capacities to neutralize oxidative stress.

There are only a few studies that compare the effects of metasilicates and silicon nanoparticles and even fewer that have analyzed the effects of these compounds on plants treated with cadmium. Rahman et al. [106] showed that in cadmium-stressed corn, both sodium metasilicate and silicon nanoparticles increased antioxidant enzyme activity and reduced Cd content in grains—with reductions of 60.6% versus 62.2% at 25 mg/kg Cd and 43.2% versus 48.7% at 50 mg/kg Cd for sodium silicate and SiNPs, respectively. On the other hand, Yan et al. [93] indicated that SiNPs are more effective in promoting tomato growth and alleviating oxidative damage than Si in Cd-stressed tomatoes by modulating the antioxidant system and reducing apoplastic Cd uptake.

The results presented above clearly show that Si application positively influences plant antioxidant systems under Cd stress. This can help Cd-stressed plants better counteract the negative effects of Cd stress. The decrease in MDA and H_2_O_2_ levels after Si application may be one of the protective effects of Si. Interestingly, these effects were observed regardless of the method of application (soil additive, foliar spray, or addition to hydroponic solution). In the case of SiNPs, the results may possibly depend on the SiNPs size. Small-diameter (3–5 nm) nanoparticles may interfere with cytoplasmic antioxidant systems; larger ones (10–20 nm) can inhibit antioxidant enzymes or lead to ROS accumulation in the apoplasm [105]. However, this mechanism requires further research. Overall, these observations indicate that Si can potentially be used as a protective agent to mitigate Cd-induced oxidative stress in plants. The enhanced antioxidant system may contribute to improved plant tolerance to Cd toxicity.

### 3.2. Metal Chelation and Compartmentalization

The evolution of plant defense mechanisms has enabled plants to reduce the uptake and bioaccumulation of toxic elements such as heavy metals in their tissues. Different plant species differ in their sensitivity and ability to survive in Cd-contaminated soils. Plants are classified into three types depending on their ability to adapt and survive in a Cd-contaminated environment: hyperaccumulators, excluders, and indicators [107,108]. Plants that are hyperaccumulators are characterized by the accumulation of large amounts of heavy metals in the vacuoles of leaf cells, in which they differ from other plants that accumulate heavy metals mainly in the vacuoles of root cells [109].

Processes leading to detoxification of the plant can occur as a result of reduced Cd transport to aerial plant parts, either through the formation of physical barriers (suberin, lignin, cellulose, silica) that inhibit apoplastic transport to the xylem or through the synthesis of sulfur-containing chelates (phytochelatins, among others) that bind and store Cd in vacuoles. Moreover, it was shown that pectins can lead to increased cadmium deposition in root cell walls, indicating the crucial importance of these polysaccharides in detoxification processes [35]. However, there are factors that influence the stimulation of plants’ natural abilities to cope with adverse environmental conditions. Numerous studies point to the effect of silicon and silicon nanoparticles on increasing the detoxification capacity of plants.

Phytochelatins (PCs) are synthesized from glutathione (GSH) in the cytosol of plant cells from which they are transported to the vacuole [109]. The presence of Cd was shown to increase the production of PCs [110,111]. It was also found that silicon can increase concentrations of phytochelatins and glutathione in plants exposed to cadmium stress. Moreover, silicon supplementation may have the effect of reducing the expression of cadmium transporters: *OsNRAMP5* and *OsHMA2* [112,113]. The OsNRAMP5 transporter was localized in the epidermis, outer cortex, and in the vicinity of the xylem tissue of the root, and it was determined to be responsible for the transport of manganese (Mn), iron (Fe), as well as cadmium (Cd) into the plant [114]. Moreover, mutation of the *OsNRAMP5* gene was found to lead to a reduction in the transport of Cd from the roots to the aerial parts of plants [115]. OsHMA2, on the other hand, was characterized as a transporter of zinc (Zn) and Cd from roots to plant shoots [116]. A study of Wei et al. [117] showed that supplementation of rice grown under cadmium stress induced by 60 μmol L^−1^ CdCl_2_ with 1 mmol L^−1^ Na_2_SiO_3_ increased the synthesis of phytochelatin 2 and 3 (PC2, PC3). It was also shown that silicon increased cadmium deposition in root cell walls, which was found mainly in pectins, where Cd content was higher compared to hemicellulose. Similar results were obtained in pea plants, where silicon supplementation with 1.8 mM H_4_O_4_Si and treatment with 20 µM CdSO_4_ increased the synthesis of the phytochelatin precursor (GSH1) compared to plants treated with cadmium. Moreover, it was suggested that the regulation of iron transporter (RIT1) expression may affect changes in Cd transport to aerial plant organs. It was also shown that the addition of Si caused the reduction in RIT1 expression in the shoots of the analyzed plants [118]. On the other hand, the application of 2 mM Na_2_SiO_3_ in *Phaseolus lunatus* plants treated with 75 mg kg^−1^ CdCl_2_ resulted in a statistically significant increase in glutathione synthesis compared to plants grown under cadmium stress alone [119]. Interestingly, in a study of Greger et al. [120], no significant differences were observed in the levels of the PC2 and PC3 and GSH in shoot protoplasts of wheat plants treated in situ for 1 h with cadmium and silicon compared to plants treated with cadmium. In contrast, significant differences were observed in plants that were grown for 4 days in the presence of either cadmium or silicon, where a statistically significant increase in PC2 content was observed in shoots of plants treated with Si + Cd compared to plants treated with Cd.

**Figure 1:**
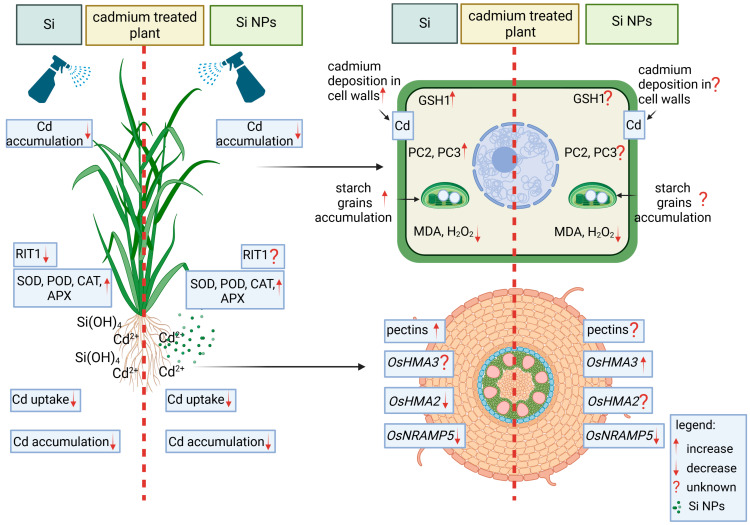
The effect of Si and Si NPs on Cd-treated plants; created in BioRender. Komorowska-Trepner, M. (2025) https://BioRender.com/by70hy1, accessed on 10 September 2025.

Cadmium uptake is affected by soil acidity, with the highest levels of uptake by rice plants occurring at a pH of 6 [121]. It was also found that *P. sativum* plants treated with 50 μM CdSO_4_ show higher Cd accumulation in roots when grown in a solution of pH = 5 compared to pH = 6 [122]. Silicon may exhibit different properties affecting the uptake of Cd by plant roots due to different soil acidities. It was noted that the application of 2 mM Na_2_SiO_3_ to plants exposed to cadmium reduced Cd content in roots of the analyzed plants (grown at pH = 5 and pH = 6) compared to plants treated with cadmium [122]. Another study showed that the addition of silicon in acidic soil (pH = 5.44) reduced cadmium uptake as a result of increased binding of the element in the soil. In contrast, the application of silicon in alkaline soil (pH = 8.15) leads to the formation of Si-Cd complexes that are not bioavailable to rice plants [71]. The effect of soluble Si-Cd complexes, which limit Cd uptake by plants, consists in the reduced content of Cd^2+^ ions in the soil solution that could be absorbed by plant roots. Guo et al. [123] showed that the above-mentioned complexes are formed from the combination of the Si-O group and C. Interestingly, it was also shown that the application forms of silicon differ in the strength of cadmium binding. Silicon occurring in the dissolved form (Si(OH)_4_) was characterized as a compound forming the strongest connections with cadmium coordination bonds. In contrast, silicon in the solid form, occurring as silica (SiO_2_), binds cadmium by adsorption. Interestingly, the adsorption reaction of Cd^2+^ ions on the silica surface can be hindered by the presence of hydration ions, i.e., water particles surrounding cadmium cations, while this phenomenon can be partially neutralized by the addition of chloride ions (Cl^−^) [124]. Figure 1 summarizes the issues presented in this paper, covering the impact of silicon and silicon nanoparticles on Cd uptake and bioaccumulation, manifested by a reduction in the expression of Cd transporters. Figure 1 also shows the effect of Si on the binding and compartmentalization of cadmium in cell walls and vacuoles of plant cells as a result of increased pectin content or phytohelatin synthesis.

### 3.3. Effects on Nutrient Uptake and Transport

Nutrient uptake by plants is crucial for the proper functioning of the physical and biochemical processes taking place in their tissues. Plants derive essential micro- and macronutrients through the root system from the soil solution. The first barrier that ions must overcome is the cell wall of root cells, which is considered low selective. This means that it does not recognize ions based on biological significance to the plant organism but instead acts as an ion exchanger. Another barrier is formed by binding sites characterized by high affinity, whose function is to transport ions across the plasma membrane [125]. Plants grown on Cd-contaminated soils are at risk of developing numerous deficiencies. Cadmium can take the place of compounds necessary for the proper functioning of the plant organism in membrane transporters of root cells. Based on analyses conducted on *Amaranthus hypochondriacus* L., it was shown that Ca^2+^ channels are most important in cadmium transport [126]. An experiment was also conducted to evaluate the effect of Cd on the movements of the stomatal apparatus of *Arabidopsis thaliana*, and it was suggested that this element may penetrate the interior of the stomatal cells through Ca^2+^ channels and affect the closure of the stomata [127]. Interestingly, it was found that individual ions can exhibit differential effects on Cd transport, for example, manganese increases cadmium uptake, while zinc inhibits it [128].

Numerous studies confirmed the effectiveness of metasilicates and silicon nanoparticles in the process of reducing cadmium transport to the aerial parts of plants, as well as their positive effect on stimulating nutrient transport. Table 2 and Table 3 summarize the effects of silicon and silicon nanoparticles, respectively, on nutrient transport in individual plant species treated with cadmium.

### 3.4. Transcriptomic and Proteomic Insights on Silicon’s Effect on Cd-Treated Plants

Recent years have witnessed a surge in the interest in the molecular mechanisms underlying plant responses to heavy metal stress, including cadmium. In particular, the effect of silicon on the molecular mechanisms of plant organisms’ coping with stress were extensively studied. The use of advanced transcriptomics and proteomics tools allows gaining an increasingly deep understanding of the effects of silicon on changes in gene expression and in protein synthesis, which are a key to understanding the defense and detoxification mechanisms of plants. In analyzing the effects of Cd and Si on plants, it is essential to assess gene expression changes of transporters of these compounds and transporters of compounds involved in detoxification. ABC transporters (ATP synthase (ATP)-binding cassette transporters) are involved in the transport of numerous hormones involved in plant stress responses (abscisic acid, salicylic acid, jasmonic acid, auxins, and gibberellins). Presumably, the above transporters may be involved in plant responses to the presence of cadmium stress. Treatment of tomato plants with Cd was shown to alter the expression of *SlABC* genes, which may indicate their involvement in the detoxification of these plants [138]. The treatment of tomato seedlings grown under cadmium stress with silicon (Na_2_SiO_3_) also increases the expression of ABC family transporter genes (*SlABCG*) [139]. In addition to ABC transporters, cadmium uptake can also occur via NRAMP (natural resistance-associated macrophage protein), HMA (heavy metal-transporting ATPases), ZIP (zinc and iron regulated transporter protein), and the YSL (yellow stripe-like) transporter family [140].

Silicon affected the transcriptome and induced numerous positive morphological and physiological changes in cadmium-treated plants. Si treatment of rice plants was shown to reduce the expression of *Nramp5* genes, which belong to Cd transporter genes. Interestingly, silicon can also increase the expression of Si transporters, i.e., Lsi1 [141]. Expression levels of Lsi1 transporters play a key role in the mechanisms of silicon’s neutralizing effect on cadmium-treated plants [142]. It was shown that SiNPs can also inhibit the expression of cadmium transporter genes (*OsNramp5*), as well as increase the expression of *OsHMA3* genes, which are responsible for the transport of Cd to the vacuole [143]. Shao et al. [144] showed that treating only half of the roots of a rice plant with silicic acid also reduced *OsNramp5* expression in half of the unaffected roots. However, Sun et al. [145] found that foliar spray application of SiO_2_NPs in cadmium-treated rice plants did not cause changes in the expression of *OsNramp5*, *OsHMA2*, *OsHMA3* genes, in opposition to the former study.

Silicon also affects the regulation of the expression of other genes responsible for sequestration and detoxification in plants. Supplementation with SiO_2_NPs contributed to changes in the expression of *Os01g0524500* and *Os06g0514800*, the genes which are likely involved in the processes of neutralizing the effects of Cd in the roots of rice [146]. In contrast, in tomato seedlings grown under cadmium stress, silicon (Na_2_SiO_3_) treatment affects the regulation of the expression of genes encoding numerous transcription factors, including WRKY, NAC, ERF, MYB, HSF, and bHLH, involved in limiting Cd accumulation in tissues [139]. Moreover, it was suggested that the regulation of iron transport may also cause the reduction in Cd transport to the aerial parts of plants. The treatment of *Pisum sativum* plants with silicon (H_4_O_4_Si) and cadmium reduced the expression of an iron transporter (*RIT1*) in the shoots of the analyzed plants compared to plants exposed to cadmium stress alone [118]. It was shown that silicon (K_2_SiO_3_) treatment of alfalfa (*Medicago sativa* L.) plants treated with Cd reduced the iron and cadmium concentration in shoots and roots compared to plants treated with cadmium alone [147]. Moreover, studies conducted on rice plants showed that the effect of SiO_2_NPs is specific to certain plant tissues [141].

The positive effect of silicon on Cd-treated plants manifests itself not only as a direct effect on the transport and distribution of this element but also as a factor influencing other important plant functions. Photosynthesis is the basic process that enables plant organisms to produce organic compounds necessary for life [148]. Supplementation of *Sedum alfredii* plants exposed to cadmium with silicon (SiO_2_) increased the expression of genes encoding LHCB (chlorophyll a-b binding protein) and PSB (photosystem I reaction center subunit) proteins, which play key roles in photosynthesis. An increase in the LHCB protein expression, which is involved in the capture of light by the chlorophyll molecule, and the PSB protein, which affects the processes taking place in photosystem II (PSII), are important in improving plant tolerance to cadmium stress [149]. Moreover, silicon (Na_2_SiO_3_·9H_2_O) supplementation can increase the expression of photosystem II-related genes: *Lhca2*, *Lhcb1*, *Lhcb2*, *Lhcb5* [150].

### 3.5. Subcellular Changes

The structure and composition of the cell wall are the primary site of subcellular alterations caused by Cd treatment in plants. On the basis of analyses carried out on *Juglans sigillata* plants, it was found that Cd + Si treatment increased the biosynthesis of cell wall components and reduced metabolites of cell energy metabolism [150]. It was shown that contact of corn roots with cadmium resulted in lignification of the cell walls of the inner part of the primary cortex and pericycle, as well as the primary elements of the xylem, which did not occur in the roots of the control sample (without the addition of Cd) [33]. It was also found that exposure of *Brassica juncea* plants to Cd caused an increase in lignin content by 10.2% and in pectin content by 30.34% in the roots of the tested plants compared to the control sample. In turn, Głowacka et al. [34] showed, using transmission electron microscope (TEM) analyses, that treatment of pea roots with lower concentrations of Cd (with values of 50 and 100 µM CdSO_4_) increased the amount of fat bodies and starch in the cells. In contrast, the application of higher concentrations of Cd (200 µM CdSO_4_) further affected the increased deposition of suberin in pea root endoderm cell walls.

Pectins are polysaccharides of plant cell walls [151], which play a key role in the process of cell adhesion. In the study of Gołębiowski et al. [35], it was shown that pectins of the pea (*Pisum sativum* L.) roots have the ability to bind Cd ions and can be important during detoxification of the plant. Increased pectin levels after silicon application were observed also in other plants, e.g., *Sedum alfredii* Hance and rice where an increase in Cd content in pectins was observed [149,152]. In addition, increased pectin methylesterase (PME) activity was observed in silicon-supplemented pea roots [35]. PME is an enzyme that catalyzes the demethylation of galacturonic acid methyl esters, which leads to an increase in the number of free carboxyl groups (-COO^−^) in pectin [153]. Increased Si-mediated PME activity can affect the integrity and rigidity of plant tissues and thereby promote Cd binding, hindering its symplastic and apoplastic transport within root tissues and further from roots to shoots. Studies by Guo et al. [154] and Yang et al. [149] confirmed that silicon also induces the expression of genes related to cell wall biosynthesis, including genes encoding PME.

It is important to note that the mechanisms limiting Cd uptake may be influenced by the age of plant roots. In *Kandelia obovata*, it was found that young and older roots differ in the type of lignin they develop. Young roots bind cadmium to the cell wall by retaining cadmium ions through chemical bonding with lignin functional groups. In contrast, older roots neutralize Cd using a blocking mechanism involving a mechanical barrier formed by thickened, lignified cell walls of the roots [155]. Radotić et al. [156] found that silicon likely inhibits the formation of large lignin fragments. However, the influence of silicon on lignification is still under investigation. In the study of Djikanović et al. [157], it was shown that low concentrations of silicon promote the aggregation of lignin oligomers into larger structures, whereas higher concentrations of Si inhibit the growth of lignin molecules by increasing the repulsion between lignin oligomers.

Cadmium may also affect cell division in plants. It was demonstrated that treatment of *Arabidopsis thaliana* plants with 5 μM CdSO_4_ resulted in a reduction in the concentration of leaf cell nuclei, as well as a decrease in the number of cells with high ploidy. Vandionant et al. [158] showed that Cd inhibits the cell cycle in *Arabidopsis thaliana*. The study also analyzed the effect of Cd on the root apical meristem of the above-mentioned plant. It was demonstrated that higher ROS levels induced by Cd in root cells lead to changes in the cortical microtubule network and disturbances in metabolic processes related to starch and sucrose, resulting in decrease in their levels [159]. The microtubule network in plant cells may also change depending on environmental conditions such as drought, salinity, or low temperatures [160]. Furthermore, microtubules play an important role in regulating the orientation of cellulose fibrils [161].

Moreover, numerous studies have also shown that both silicon and cadmium can undergo bioaccumulation within plant cell structures. Asgari et al. [162] revealed that both silicon oxide nanoparticles and silicon applied in ionic form are deposited in the cell walls of parenchyma cells. TEM analysis of *Monochoria hastata* treated with Cd showed the presence of electron-dense material in the cell walls, vacuoles, chloroplasts, and mitochondria of the examined plants [163]. It was also demonstrated that foliar application of SNPs in *Brassica napus* plants exposed to cadmium leads to elevated deposition of Cd in the cell walls of the roots [164].

The numerous changes resulting from Cd treatment in plants mentioned above are the outcome of natural plant mechanisms for coping with stress. However, Si supplementation can influence the modification of these responses. It has been shown that short-term Cd treatment (6 h) of *Triticum aestivum* plants grown in nutrient solution supplemented with Si (Na_2_SiO_3_) reduces cadmium content in the roots due to delayed formation of the suberin barrier. This phenomenon leads to an increased Cd content in the shoots. In contrast, long-term Cd treatment (7 days) promotes root cell wall suberization, which limits Cd transport to the aerial parts [165]. Additionally, it was found that cadmium stress in *Pisum sativum* plants leads to a reduction in root diameter and stomatal surface area in leaves. However, foliar application of Si contributes to an increase in stomatal surface area compared to Cd-treated plants [98].

The above findings indicate the effects of Si, SiNPs, and Cd at the cellular and subcellular levels. Silicon supplementation in plants exposed to cadmium stress supports the plant’s natural defense mechanisms and improves their tolerance to abiotic stress conditions. Interestingly, the effect of Si is often dependent on the duration of plant exposure to cadmium.

## 4. Future Perspectives

Cadmium contamination of soils currently constitutes a serious environmental issue. Various forms of industry as well as phosphate fertilizers used in agriculture represent the main sources of this element’s deposition in arable soils. This leads to many severe consequences that affect numerous organisms within the food chain—from Cd-contaminated plants to animals and humans [166]. The present paper summarizes the current knowledge regarding the impact of silicon on plants exposed to cadmium stress. Numerous studies have confirmed that silicon can contribute to the inhibition of Cd uptake by reducing the expression of *OsNramp5* transporters [143,144]. However, there are also findings that contradict this statement, suggesting that the reduction in transporter expression may result from other substances present in the soil or in the used culture solution rather than from the direct action of silicon [145]. It is crucial to conduct further research in order to provide more data and expand the understanding of how silicon influences Cd transporter(s) expression.

Moreover, there are conflicting theories regarding the effects of silicon on plant organisms. These contradictions arise from interspecies differences in the ability of plants to absorb and bioaccumulate this element [50]. Furthermore, numerous studies indicate changes in nutrient uptake resulting from silicon supplementation in plants exposed to cadmium stress; however, these findings vary depending on the analyzed plant species [81,84,129]. It is essential to conduct further analyses that will provide data for a greater number of species, as this will allow broader interspecies comparisons of silicon’s effects on Cd-treated plants.

Nevertheless, it should be emphasized that all studies have indicated a positive effect of silicon on plants under cadmium stress, manifested itself in subcellular changes, increased antioxidant enzyme activity, and reduced levels of MDA and H_2_O_2_ [97,99,161]. Si contributed to an increase in pectin content, which has the ability to bind Cd and thus detoxify the plant organism [35]. It was also found that silicon supplementation in plants not exposed to cadmium stress did not lead to significant changes in physiological parameters [167]. Therefore, Si supplementation is particularly important in soils from highly industrialized and polluted areas. Both silicon and silicon nanoparticles are considered as potential substances with significant applicability in agriculture. Nanoparticles of silicon, one of the most abundant elements in the world, are regarded as relatively non-toxic and safe (GRAS). Furthermore, nanoparticle size plays a key role in their toxicity, and the small size of silicon nanoparticles makes them relatively non-toxic in nature; therefore, their use is more environmentally friendly [168]. Their easy application techniques, such as seed priming or spraying, appear to be an innovative approach that enables plant protection against abiotic stress, including the presence of Cd in soil. However, further research is necessary to confirm the effectiveness of these methods in coping with environmental and soil contamination.

Production of Si nanoparticles involves both biological and synthetic methods. Synthetic methods can have an adverse impact on the natural environment. On the other hand, the biological approach to Si NPs production is a promising development because it uses biological waste. The waste for Si NPs production comprises ash from agricultural waste, or waste from plant production, e.g., rice straw and husks. This enables simultaneous recycling of waste and nanoparticle production. Therefore, conducting research on the effect of Si NPs is extremely important, as it can enhance the growth of agriculture while protecting the natural environment owing to waste neutralization [168]. Moreover, application of Si NPs is likely to contribute to the reduction in the use of artificial fertilizers and pesticides in agriculture. Nonetheless, it is necessary to continue studies that will allow researchers to evaluate long-term effects of this process [169].

This review summarized the up-to-date knowledge on the effects of silicon compounds on plants exposed to cadmium stress and implicates the scarcity of research papers focusing on the comparison of the impact of Si versus Si NPs on Cd-treated plants. It is crucial to continue further studies in this regard in order to obtain detailed data, which will help to develop farming and protect nature. Furthermore, should Si NPs be determined to have a stronger or more targeted effect than conventional Si on neutralization of Cd influence on plants, it could contribute to the development of more eco-friendly agricultural strategies. It appears to be possible because, as mentioned before, Si NPs are thought to be non-toxic and safe. Equally important is to carry out studies with the use of advanced molecular methods, which should allow researchers to make more complete analyses of the response of S- or Si NPs-treated plants to the presence of Cd. Moreover, comparative analysis of the influence of Si and Si NPs should cover a wide range of species in order to obtain reliable data. Studies should also focus on the evaluation of the impact of these compounds on changes in the plant uptake of elements essential for the proper functioning of plants and on subcellular changes, including pectin deposition and lignification of cel walls, induced by the application of silicon. For a more complete comparison of changes induced by Si and Si NPs on plants under cadmium stress, it is necessary to obtain such information.

**Figure 2:**
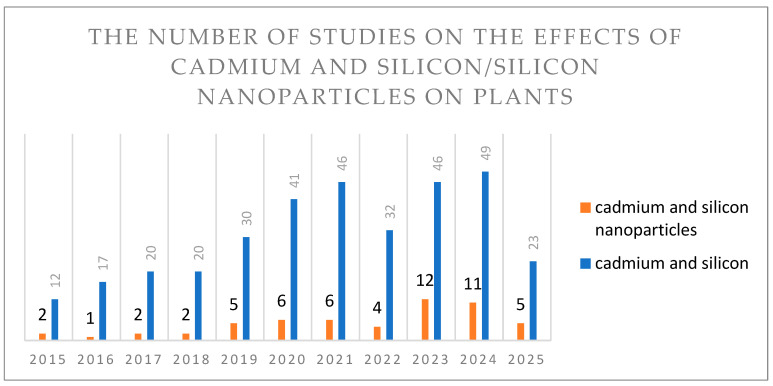
Number of publications on the effects of cadmium and silicon/silicon nanoparticles on plants between 2015 and 2025.

To analyze the number of publications concerning the effects of cadmium and silicon or silicon nanoparticles on plant organisms between 2015 and 2025, the PubMed database was used. The search was conducted using the following keywords: “cadmium AND silicon AND plant”, which yielded 336 scientific publications from 2015–2025, and “cadmium AND silicon nanoparticles AND plant”, which yielded 56 scientific publications from 2015–2025 (Figure 2). It was found that the number of publications assessing the effects of silicon nanoparticles on cadmium-treated plants was 83% lower compared to those focusing on the effects of silicon.

**Figure 3:**
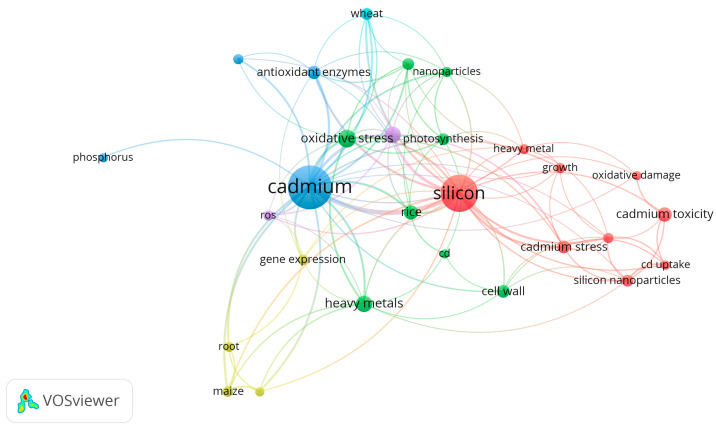
Keyword co-occurrence map generated using VOSviewer based on 154 publications related to the effects of cadmium (Cd) and silicon (Si) on plants. Colors represent distinct thematic clusters, node size indicates keyword frequency, and connecting lines illustrate the strength of co-occurrence among terms.

To perform the bibliometric analysis of the publications presented in this study, the VOSviewer tool (version 1.6.20) was used, which allows the visualization of keyword co-occurrence networks. A total of 154 bibliographic records were retrieved from the Google Scholar database and saved in RIS format using the Mendeley program (Version 2.135.0). A keyword co-occurrence analysis was carried out by setting the minimum number of occurrences of a given keyword to five. After applying the criteria, a total of 28 keywords met the conditions and were included in the analysis (Figure 3).

The bibliometric analysis revealed five main thematic clusters related to research on the impact of cadmium and silicon on plant organisms. The blue cluster, dominated by the term “*cadmium*”, focuses on the toxic effects of cadmium, with particular emphasis on oxidative stress, antioxidant enzymes, and the model organism *A. thaliana*. The red cluster (“*silicon*”) centers on the role of silicon in alleviating stress induced by cadmium, encompassing terms such as “*cadmium toxicity*”, “*oxidative damage*”, and “*Cd uptake*”. The green cluster includes terms related to photosynthesis, reactive oxygen species, and the effects of Cd and Si on plant physiological parameters. The purple cluster covers general oxidative stress processes and responses to stress factors, linking both Cd and Si with ROS and signaling processes. The yellow cluster relates to the localization of Cd in plant tissues. The presented co-occurrence map indicates strong connections between cadmium and oxidative stress, as well as between silicon and its protective role in plants subjected to cadmium stress.

## 5. Conclusions

In summary, the key comparative effects of conventional Si and SiNPs identified in this review are as follows:

- Reduction in Cd uptake, translocation, and accumulation in plants.
- Si decreases the bioavailability of Cd to plant organisms by the increase of soil pH [70].
- Si decreases the expression of Cd transporters, *OsNRAMP5* and *OsHMA2,* in rice [112,113].
- There are inconsistent data on the effect of SiNPs on the expression of Cd transporters. It has been found that SiNPs may inhibit the expression of *OsNramp5* and increase the expression of *OsHMA3* [143]; there are also data indicating that SiO_2_NPs have no effect on the expression of the *OsNramp5*, *OsHMA2*, and *OsHMA3* genes [145].
- Si/SiNPs decreases accumulation of Cd in roots and shoots.
- Induction of protective mechanisms in plants.
- Si/SiNPs increase the activity of antioxidant enzymes, SOD, POD, CAT, and APX, compared to plants exposed to Cd stress [69,73,83,89,90]. However, the effect of SiNPs may depend on particle size [105].
- Si application may influence the increase in phytochelatin synthesis: PC2 and PC3 [117]. There are no data on SiNPs on phytochelatin synthesis.
- The application of Si may increase the pectin content in the cell walls of root cells, which can bind Cd and limit its translocation to above-ground parts [35]. There are no data on SiNPs on pectin content.
- Potential role of conventional Si and SiNPs in sustainable agriculture.

Both Si and SiNPs can alleviate Cd-induced stress in plants and are recognized as safe for agricultural applications. However, the potential and mechanisms of SiNPs action need further investigation.

## Figures and Tables

**Table 1:** Cadmium effect on plant physiology.

Species	Cultivation	Concentration of the Cd Salt Used	Concentration of Cd^2+^ in Salt	Results	References
*Pisum sativum* (L.)	Pots with peat substrate	6 mM CdSO_4_	674.5 mg L^−1^	leaf growth phase: - stomata closed, - intercellular CO_2_ concentration ↓, - transpiration rate ↓, - dry matter ↓, - chlorophyll content ↓	[26]
*Pisum sativum* (L.)	Pots with reconstituted sand, supplemented with vermicompost	400 µM CdCl_2_	44.96 mg L^−1^	- dry weight and fresh weight ↓, - shoot length and root length ↓, - chlorophyll *a*, chlorophyll *b*, and carotenoid content ↓ - proline and H_2_O_2_ ↑.	[27]
*Pisum sativum* (L.)	Pots with sterilized soil	10 mg kg^−1^ Cd	10 mg kg^−1^ Cd	- average plant height ↓, - number of leaves and leaf area ↓, - number of flowers, seeds, and fruits ↓.	[28]
*Pisum sativum* (L.)	Petri dishes filled with distilled water and streptomycin sulfate	1 mM CdCl_2_	112.41 mg L^−1^	- germination ↓−20%, - respiratory activity ↓, - β-amylase activity ↓, - dry matter ↓.	[29]
*Pisum sativum* L.	Pots with perlite	200 µM CdSO_4_	22.48 mg L^−1^	- delayed flowering, - number of flowers ↑ (stress-induced flowering), - number of mature pods ↓, - the ratio of internode length to shoot length ↑.	[30]
*Arabidopsis thaliana*	Mineral wool saturated with a 1:10 Hoagland solution containing half the standard dose of iron in the form of Fe-EDTA and microelements.	10 mM CdSO_4_	1124.1 mg L^−1^	- mRNA levels of genes involved in phytochelatin synthesis ↑, - loss of cellular redox homeostasis.	[31]
*Triticum aestivum* L.	Polyethylene pots filled with modified Hoagland nutrient solution	1 mM Cd(NO_3_)_2_	112.4 mg L^−1^	- plant growth ↓, - chlorophyll content ↓, - photosynthesis ↓, - disruption of chloroplast structure—expansion of thylakoid membranes.	[32]

Note: “↑”: increase; “↓”: decrease.

**Table 2:** Effect of silicon metasilicates on uptake and transport of Cd and micro- and macronutrients in plants.

Species	Cultivation	Cd Salt Concentration	Concentration and Type of Si	Method of Application Si	Cd Effect	Impact of Cd + Si	Si Impact on Cd Accumulation	References
*Pisum sativum* (L.)	Growing in pots	150 mg L^−1^ CdSO_4_·8H_2_O	2 mM Na_2_SiO_3_	Si applied with nutrient solution	**Shoots:** ↓ S (34.69%), Mg (58.33%), Ca (43.47%), P (48.62%), K (57.55%), B (45.00%), Cu (28.48%), Fe (27.05%), Mn (56.07%), Zn (37.85%) vs. control plants.	**Shoots:** S, Mg, Ca, P, K, B, Cu, Fe, Mn and Zn in shoots: - ↑ vs. Cd-treated plants, - ↓ vs. control plants.	**Roots, shoots, leaves:** ↓ Cd in vs. Cd-treated plants.	[84]
*Phoenix dactylifera* L.	Growing in pots	200 μM Cd	1.0 mM Na_2_SiO_3_	Applied to the root zone	**Roots:** ↓ K, Mg vs. control; Ca—ns (not significant) vs. control plants. **Shoots (Cd-treated):** ↑ K, P, Mg, Ca vs. control plants.	**Roots:** ↑ K, Mg, Ca and ↓ P vs. Cd-treated plants **Shoots:** ↑ K, Mg and Ca and P—ns (not significant) vs. Cd-treated plants.	**Roots and shoots:** ↓ Cd in vs. Cd-treated plants.	[129]
*Triticum aestivum* L.	Growing in pots	200 µM CdSO_4_·8H_2_O	1 mM Na_2_O_3_Si·9H_2_O	Si applied with nutrient solution	**Roots**: ↓: K, Ca, Mg, P, Fe, Cu, Mn, Si vs. control plants; ↑ Zn vs. control plants. **Shoots**: ↓: K, Ca, Mg, P, Fe, Zn, Cu, Mn, Si vs. control plants.	**Roots:** ↑ Ca, Mg, P, Fe, Si; ↓ Zn, Cu; K, Mn—ns (not significant) vs. Cd-treated plants **Shoots:** ↑ Ca, Mg, P, Fe, Zn, Cu, Mn, Si; K—ns (not significant) vs. Cd-treated plants	**Roots and shoots:** ↓ Cd in vs. Cd-treated plants.	[81]
*Solanum lycopersium* Mill	Hydroponic cultivation	3 mg L^−1^ CdCl_2_·2.5H_2_O	3 mmol L^−1^ Na_2_O_3_Si·9H_2_O	Si applied with nutrient solution	-	**Roots: ↑** Fe, Mn, Zn; K, Ca, Mg—ns (not significant) vs. Cd-treated plants **Shoots: ↑:** K, Ca, Mg, Fe, Mn, Zn vs. Cd-treated plants	**Roots and shoots:** ↓ Cd in vs. Cd-treated plants.	[130]
*Triticum turgidum* L. cv. Claudio	Hydroponic cultivation	50 μM Cd(NO_3_)_2_·4H_2_O	1 mM Si(KOH)_2_	Si applied with nutrient solution	**Roots:** ↓ Mn; Zn—ns (not significant) vs. control plants **Shoots:** ↓: Mn, Zn vs. control plants	**Roots: ↑** Zn; Mn—ns (not significant) vs. Cd-treated plants **Shoots:** Mn, Zn—ns (not significant) vs. Cd-treated plants	**Roots:** Cd—ns (not significant) vs. Cd-treated plants **Shoots:** ↓ Cd in vs. Cd-treated plants.	[131]
*Triticum aestivum* L.	Hydroponic cultivation	200 μmol L^−1^ CdCl_2_	3 mmol L^−1^ Na_2_SiO_3_	Si applied with nutrient solution	**Roots:** ↓: N, P, K, Ca, Mg, Zn vs. control plants	**Roots: ↑** N, P, K, Ca, Mg, Zn vs. Cd-treated plants.	**Roots and shoots:** ↓ Cd in vs. Cd-treated plants.	[90]
*Vigna unguiculata* (L.) Walp.	Growing in pots, semi-hydroponic	500 μM CdCl_2_	2.50 mM Na_2_SiO_3_·9H_2_O	Si applied with nutrient solution	**Roots, stems and leaves:** ↓: P, K, Ca, Mg, S vs. control plants	**Roots, stems and leaves: ↑** K, Ca, Mg, S vs. Cd-treated plants.	**Roots, stems and leaves:** ↓ Cd in vs. Cd-treated plants.	[87]
*Zea mays* L.	Growing in pots	10 mg kg^−1^ CdCl_2_ H_2_O	300 mg kg^−1^ Si in the form of fertilizer	Si mixed with soil	-	**Aboveground plant parts:** a tendency to increase the nutrient content vs. Cd-treated plants.	**Roots:** Soil-pH = 4.6, **↑** Cd; Soil pH = 6.5↓ Cd vs. Cd-treated plants. **Shoots:** ↓ Cd vs. Cd-treated plants.	[132]
*Cucumis melo*	Growing in pots	200 mg kg^−1^ CdSO_4_	200 mg kg^−1^ SiO_2_	Si mixed with soil	**Leaves**: **↑**: Na, K, Fe, Ca; ↓ Mg vs. control plants	**Leaves**: **↑** Na, K, Mg, Fe, Ca vs. Si-treated plants.; ↓ Na, K, Mg, Fe, Ca vs. Cd-treated plants.	-	[133]
*Triticum aestivum* L.	Hydroponic cultivation	10 μM CdCl_2_·2.5H_2_O	1 mM Na_2_SiO_3_·9H_2_O	Si added to the nutrient solution	**Roots:** ↓ P, Na Mn; Ca, B, Zn, Mg, K—ns (not significant) vs. control plants **Shoots:** ↓ B; P, Ca, Si, K, Mg, Na, Mn, Cu, Al, Zn—ns (not significant) vs. control plants	**Roots:** ↓ P, Ca, S, Fe, B, Mo, K, Mg, Cu; **↑:** Mn, Si vs. Cd-treated plants. **Shoots:** ↓ Ca, S, B, Mo; P, Fe, Mg, Cu, Al, Zn—ns (not significant) vs. Cd-treated plants.	**Roots and shoots:** ↓ Cd in vs. Cd-treated plants.	[134]

Note: “↑”: increase; “↓”: decrease.

**Table 3:** Effect of silicon nanoparticles on uptake and transport of Cd and micro- and macronutrients in plants.

Species	Cultivation	Cd Salt Concentration	Concentration and Type of SiNPs	Method of Application SiNPs	Cd Effect	Impact of Cd + SiNPs	SiNPs Impact on Cd Accumulation	References
*Triticum aestivum* L.	Cultivation in a field contaminated with Cd	4.23 mg kg^−1^ Cd	300 mg L^−1^ Si NPs	Si NPs applied as a foliar spray	-	**Grain unit:** ↓ Fe; Zn—ns (not significant) vs. Cd-treated plants.	**Grain and straw: ↓** Cd vs. Cd-treated plants.	[135]
*Phaseolus vulgaris*	Growing in pots	2 mM CdCl_2_	20 mg L^−1^ Si NPs	Seed treatment	-	**Plant sample**: ↓ Mo, Ca, K, Mn vs. control plants.	-	[136]
*Oryza sativa* L.	Cultivation in a field contaminated with Cd	0.69 mg kg^−1^ Cd in soil	25 mM SiNPs	Foliar application	-	**Grains: ↑** K, Mg, Fe; Ca, Mn, Zn—ns (not significant) vs. Cd-treated plants. **Rachises: ↑** K, Mg, Fe; Ca Mn, Zn—ns (not significant) vs. Cd-treated plants.	**Grains and rachises: ↓** Cd vs. SiNPs -treated plants.	[137]
*Oryza sativa* L.	Hydroponic cultivation	20 µM L^−1^ CdN_2_O_6_·4H_2_O	100 mg L^−1^ SiO NPs	SiO NPs added to the nutrient solution	**Roots:** ↓ Mg, Ca and K vs. control plants **Shoots:** ↓ Mg, Ca, K, Si vs. control plants	**Roots: ↑** Mg, Ca, K, Si vs. Cd-treated plants. **Shoots: ↑** Mg, Ca, K, Si vs. Cd-treated plants.	**Roots and shoots:** ↓ Cd in vs. Cd-treated plants.	[95]

Note: “↑”: increase; “↓”: decrease.

## Data Availability

No new data were created or analyzed in this study.
